# Synergistic effect of perovskites and nitrogen-doped carbon hybrid materials for improving oxygen reduction reaction

**DOI:** 10.1038/s41598-023-47304-4

**Published:** 2023-11-13

**Authors:** R. Rohib, Saeed Ur Rehman, Eunjik Lee, Changki Kim, Hyunjoon Lee, Seung-Bok Lee, Gu-Gon Park

**Affiliations:** 1 Fuel Cell Laboratory, Korea Institute of Energy Research (KIER), 152 Gajeong-ro, Yuseoung-gu, Daejeon, 34129 Republic of Korea; 2 Department of Energy Engineering, University of Science and Technology, 217 Gajeong-ro, Yuseong-gu, Daejeon, 34113 Republic of Korea

**Keywords:** Fuel cells, Synthesis and processing

## Abstract

A fundamental understanding of the electrochemical behavior of hybrid perovskite and nitrogen-doped (N-doped) carbon is essential for the development of perovskite-based electrocatalysts in various sustainable energy device applications. In particular, the selection and modification of suitable carbon support are important for enhancing the oxygen reduction reaction (ORR) of non-platinum group metal electrocatalysts in fuel cells. Herein, we address hybrid materials composed of three representative N-doped carbon supports (BP-2000, Vulcan XC-72 and P-CNF) with valid surface areas and different series of single, double and triple perovskites: Ba_0.5_Sr_0.5_Co_0.8_Fe_0.2_O_3−δ_, (Pr_0.5_Ba_0.5_)CoO_3−δ_, and Nd_1.5_Ba_1.5_CoFeMnO_9−δ_ (NBCFM), respectively. The combination of NBCFM and N-doped BP-2000 produces a half-wave potential of 0.74 V and a current density of 5.42 mA cm^−2^ at 0.5 V versus reversible hydrogen electrode, comparable to those of the commercial Pt/C electrocatalyst (0.76 V, 5.21 mA cm^−2^). Based on physicochemical and electrochemical analyses, we have confirmed a significant improvement in the catalytic performance of low-conductivity perovskite catalyst in the ORR when nitrogen-doped carbon with enhanced electrical conductivity is introduced. Furthermore, it has been observed that nitrogen dopants play active sites, contributing to additional performance enhancement when hybridized with perovskite.

## Introduction

Fuel cells (FCs) are promising sustainable energy conversion devices owing to their high-power density, high efficiency and environmental friendliness. However, as a critical component for the oxygen reduction reaction (ORR) in the cathode, Pt-based electrocatalysts suffer from high cost, limited availability, and insufficient stability^1^. To overcome these problems, the development of a low-cost, high-performance non-platinum group metal (NPGM) nanomaterials have been investigated as an electrocatalyst in alkaline media. Numerous NPGMs have been developed, including organometallic complexes^2^, metal chalcogenides^3^, carbon-based electrocatalysts^4–6^, and iron-cobalt-based macrocyclic complexes^7,8^. However, most NPGMs have high overpotential, sluggish ORR kinetics, and are unstable compared with Pt-based electrocatalysts. Among the various NPGMs, perovskite materials (ABO_3_, where A is an alkaline or rare earth metal and B is a transition metal) have been extensively studied owing to their excellent intrinsic catalytic activity comparable with that of commercial Pt/C electrocatalysts. The unique character of perovskites, in which the structure can be modified by the partial substitution of the A or B sites, ensures these materials have different physical and electrochemical properties. In this regard, different series of single^9^, double^10^, triple^11^, and Ruddlesden-proper (RP)^12^ perovskites have been reported as effective electrocatalysts for ORR.

Ba_0.5_Sr_0.5_Co_0.8_Fe_0.2_O_3−δ_ (BSCF) is one of the original single perovskite-type with the highest catalytic performance among other single perovskites^9^. (Pr_0.5_Ba_0.5_)CoO_3−δ_ (PBCO) is a double perovskite with a pseudocubic structure and contains more oxygen vacancies than BSCF. Recently, a triple perovskite Nd_1.5_Ba_1.5_CoFeMnO_9-δ_ (NBCFM) was discovered, and it exhibited the best catalytic performance of perovskites; thus far, the performance was also superior to those of single and double perovskites^11^.

It should be noted that the factors determining the electrocatalytic performance of perovskites remain unclear. Kim et al.^11^, who first reported the investigation of the excellent catalytic activity of NBCFM, explained that the superior performance was due to the structural defects of perovskite. Therefore, the authors concluded that structural design is an effective way to improve electrocatalytic performance. However, the fact that they used approximately 30 wt% nitrogen-doped reduced graphene oxide (N-rGO) as the hybrid material was unaccounted for in the catalytic performance. In addition, other investigations using nitrogen-doped (N-doped) graphene to accelerate ORR performance should be considered as a separate factor of the hybrid perovskite-carbon material^13,14^.

It has been understood that the poor electrical conductivity of perovskites at low temperatures causes their poor electrocatalytic performance. Therefore, a hybrid of perovskite and carbon has been developed to enhance the electrical conductivity properties. The addition of a certain amount of carbon has been shown to improve the electrical conductivity of insulating materials, such as polymers and metal oxide catalysts^15^. In addition, N-doped carbon has been shown to significantly improve the catalytic performance of ORR owing to its redox-active nitrogen species^4^. Therefore, the precise role of carbon in the design of hybrid perovskite-carbon materials should be established to fully understand the actual performance of these electrocatalytic materials.

Inspired by the unique interaction between perovskite and carbon materials, where carbon plays a critical role in these material designs, we hybridized BSCF, PBCO, and NBCFM with different carbon materials as support materials. Instead of using N-doped graphene, which has some disadvantages such as its relatively high price, complicated fabrication process, and clarity problem^16–19^, we used N-doped commercial carbon, namely P-CNF, VXC-72 and BP-2000. These carbon materials are more affordable and less complicated to synthesize; they do not have contamination problems, unlike graphene. In addition, these types of carbon materials also have significant differences in intrinsic properties, such as specific surface area, electrical conductivity, and particle size. Thus, these carbon materials are suitable as perovskite tandem materials and can be sufficiently distinguished from the perovskite for the in-depth identification of the catalytic active sites of hybrid perovskite-carbon. Therefore, an investigation to understand the catalytic performance of hybrid perovskite-carbon will be more easily undertaken.

Herein, we report the evaluation of the electrochemical performance of different properties of perovskite and the significant effect of manipulating N-doped carbon on hybrid perovskite-carbon. Heat treatment in a N_2_–NH_3_ mixed gas environment was performed to prepare N-doped carbon. Different amounts of nitrogen atoms on different types of carbon materials were prepared to analyze the synergistic effect of N-doped carbon and perovskite. Electrochemical analysis was performed using a rotating disk electrode (RDE) to evaluate the ORR performance under alkaline conditions. The materials were characterized to correlate the electrochemical performance with the physicochemical properties.

## Materials and methods

### Synthesis of perovskite materials

The perovskite materials were synthesized using the Pechini method with metal-nitrate salt-based precursors such as Sr(NO_3_)_2_, Ba(NO_3_)_2_, Co(NO_3_)_3_·6H_2_O, Fe(NO_3_)_3_·9H_2_O, Pr(NO_3_)_3_·6H_2_O, Mn(NO_3_)_2_·xH_2_O, and Nd(NO_3_)_3_·6H_2_O (Sigma-Aldrich). First, the nitrate salt was dissolved in deionized water, then citric acid (CA) and ethylene glycol (EG) were added in a weight ratio of metal: CA: EG = 1: 2: 4. This was followed by heating at 70 °C until the gel dried. The gel was pre-calcined sequentially at 250 and 600 °C, resulting in a black powder. The powder was ball-milled at 2500 rpm for 24 h. The final product, pure phase perovskite, was obtained after calcination at different temperatures ranging from 700 to 1000 °C for BSCF and PBCO; for NBCFM, the powder was heat treated at 800 to 1300 °C under air. The synthesized perovskite powder was loaded into a zirconium reactor along with ten zirconia balls, each with a 5 mm diameter. The material underwent high-energy ball milling for a duration of 1 h at 450 rpm using a planetary mill (Instrument: PULVERISETTE 7 Micro Mill, FRITSCH, Germany).

### Preparation of N-doped carbon materials

Three types of carbon materials with different characteristics, platelet carbon nanofiber (P-CNF) (Vinatech Co.), Vulcan XC-72 (VXC-72, Cabot Co.), and black pearls-2000 (BP-2000, Cabot Co.), were used for the nitriding process. 1 g of each carbon material was heat treated in a high-pressure furnace at 900 °C for 1 h, at a pressure of 10 bar, and the heating rate was 7.5 °C min^−1^. The N_2_–NH_3_ mixed gas (5.01 mol% NH_3_ and N_2_ balance), used as the nitrogen source, flowed continuously at 100 cc min^−1^. The carbon was designated as N-doped carbon after the nitriding process.

### Characterization

X-ray diffraction (XRD) (Rigaku DMAX-2500, Cu Kα source, *λ* = 1.5406 Å) was performed to study the crystalline structure of the perovskite in the 2θ range of 10°–80° at a scan rate of 5° min^−1^. The specific surface area and pore volume distribution of all materials were measured using the Brauer-Emmett Teller (BET) equation (Micromeritics ASAP 2020). The morphology of the perovskites was observed using scanning electron microscope (SEM) (Instrument: Hitachi High-Tech, S-4700 FE-SEM, Japan) before and after the ball milling. A transmission electron microscopy (TEM) (Instrument: JEOL 300 kV, Tecnai F30S—Twin) was used to analyze the structure, and the elemental distribution was determined by energy dispersive X-ray spectroscopy (EDS). Furthermore, an aliquot of a mixed solution of perovskite and carbon was used to observe the adhesive interaction of the triple boundary phase using TEM.

The powder conductivity of the carbon and perovskite materials was measured using an electrical conductivity measurement tester (ECT-200K HUBIS). The apparatus consists of two 7 mm diameter steel rods placed on the upper and lower sides of a thick-walled Teflon mold. The upper rod was automatically moved down by a compressive force of 450 bar, and the sample was compressed to 3–5 mm. The elemental composition of the N-doped carbon was measured by elemental analysis (EA) (FlashEA 1112, Thermo Finnigan, USA). X-ray photoelectron spectroscopy (XPS) (Thermo VG Scientific) was performed using monochromatic Al Kα radiation to investigate the chemical bonding state of the N-doped carbon.

### Electrochemical analysis

Electrochemical measurements were performed in an RDE with a Biologic SP-240 potentiostat using a three-compartment electrochemical cell. A Pt wire was used as the counter electrode, and a reversible hydrogen electrode (RHE) (Gaskatel GmbH, Germany) was used as the reference electrode. A glassy carbon (GC) electrode (5 mm diameter, 0.196 cm^2^ area) was used as the working electrode. Before adding the catalyst ink dropwise, the electrode was polished with 0.5 µm alumina (Bohler), washed with sulfuric acid, and washed again with deionized water in a sonication bath. Catalyst materials (5 mg) were mixed with 20 µL of Nafion 5 wt% (D521, DuPont™) and a mixture of H_2_O and isopropyl alcohol (IPA) (1:9, v/v) (0.5 mL), followed by ice bath sonication for 15 min. The carbon content (pure and N-doped) was set to 20 wt% of the total ink solution for the hybrid perovskite-carbon sample. The loading targets for the pure carbon, pure perovskite, and hybrid perovskite-carbon samples were adjusted to 80 µg cm^−2^, 320 µg cm^−2^, and 400 µg cm^−2^, respectively. An aliquot of the catalyst ink was added dropwise onto the GC electrode. The cyclic voltammograms (CVs) were obtained at a scan rate of 20 mV s^−1^ from 0.03 to 1.1 V in N_2_-saturated 0.1 M KOH, and the linear sweep voltammograms (LSVs) for ORR were recorded at a scan rate of 10 mV s^−1^, 1600 rpm from 0.0 to 1.1 V in an O_2_-saturated. The ohmic resistance was measured for each RDE experiment, and the LSV curves of the samples were corrected by iR-compensation and removal of the background capacitance current.

## Results and discussion

**Figure 1:**
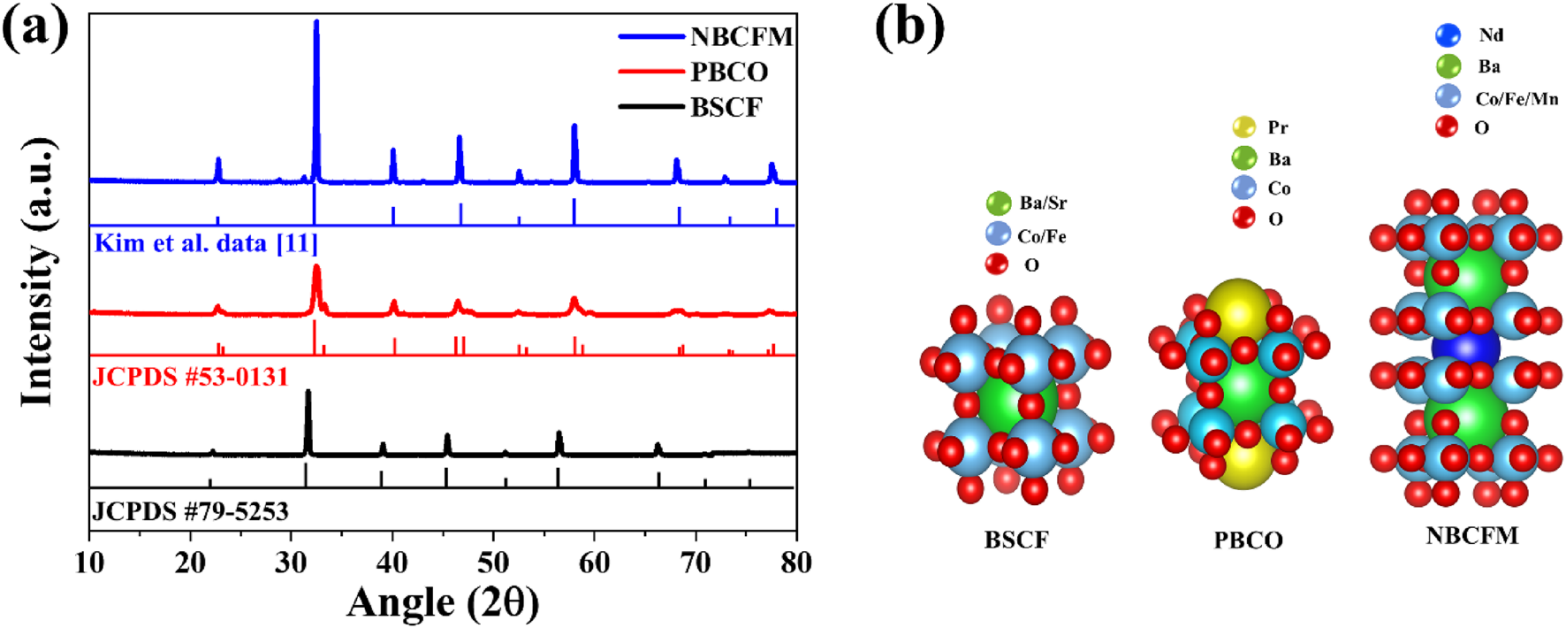
(**a**) XRD pattern and its reference peak position from JCPDS database (black and red line) and previous study (blue line). (**b**) Crystal structure of BSCF, PBCO, and NBCFM.

The crystal structures of BSCF, PBCO, and NBCFM that were synthesized by the sol–gel Pechini method were confirmed by XRD analysis. As shown in Fig. 1a., the matching process with the reference from a previous study^11^ and the JCPDS database of No.79-5253 (BSCF) and No. 53-0131 (PBCO) showed that all the peaks present prove a perovskite structure, and none of the peaks arises from another phase. Calcination was performed under different temperature conditions to obtain a pure perovskite phase. Pure and stable phases of BSCF and PBCO were obtained at 1000 °C, whereas NBCFM was obtained at 1300 °C. The change in the XRD pattern of each perovskite with annealing temperature is shown in Fig. S1. The space-filling style of the crystal structure of all perovskite materials is shown in Fig. 1b. BSCF has a cubic structure that can accommodate a wide range of oxygen nonstoichiometries without structural modification. BSCF has a moderate surface oxygen interaction energy, which causes it to be neither weak nor strong in the adsorption of O_2_; thus, it is located at the top of the volcano plot with the σ^*^-orbital (e_.g._ filling) value ≈ 1.2^20^. Pseudocubic PBCO, comprising rich oxygen vacancies concentrated in the Pr plane, forms because of the considerable difference in the ionic radius and polarizability between praseodymium and barium atoms^10^. Kim et al.^11^ reported that the modification of lanthanide or B-site atoms in NBCFM effectively changes the structural lattice distortion in the perovskite system, which lowers the charge resistance, causes lower covalency between O 2*p* and Co 3*d* on the structure, and increases the number of oxygen vacancies.

Ball milling was performed to maximize the active sites of perovskite. Figure S2 shows the SEM images of BSCF, PBCO, and NBCFM before and after ball milling. An agglomerated structure of micrometer size is clearly observed prior to ball milling; the agglomerate is successfully reduced by the ball milling process. This structure produces the usual BET surface area of perovskite, as shown in Fig. S3. The BET surface areas of BSCF, PBCO, and NBCFM are 8.91, 7.76, and 5.52 m^2^ g^−1^, respectively, after ball milling.

**Figure 2:**
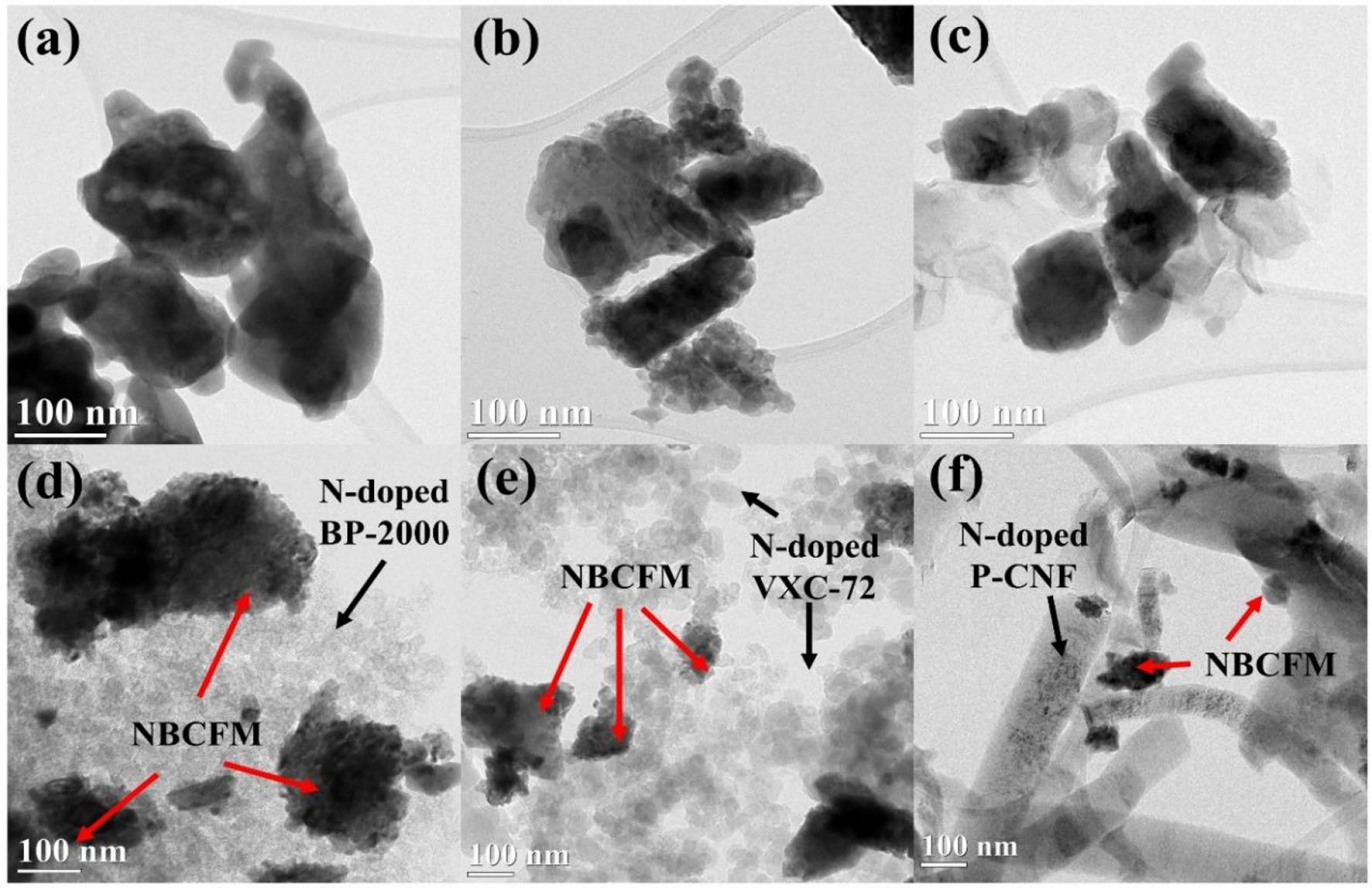
TEM images of ball milled perovskite materials (**a**) BSCF, (**b**) PBCO, (**c**) NBCFM, (**d**) NBCFM/N-BP-2000, (**e**) NBCFM/N-VXC-72, and (**f**) NBCFM/N-P-CNF sample.

**Figure 3:**
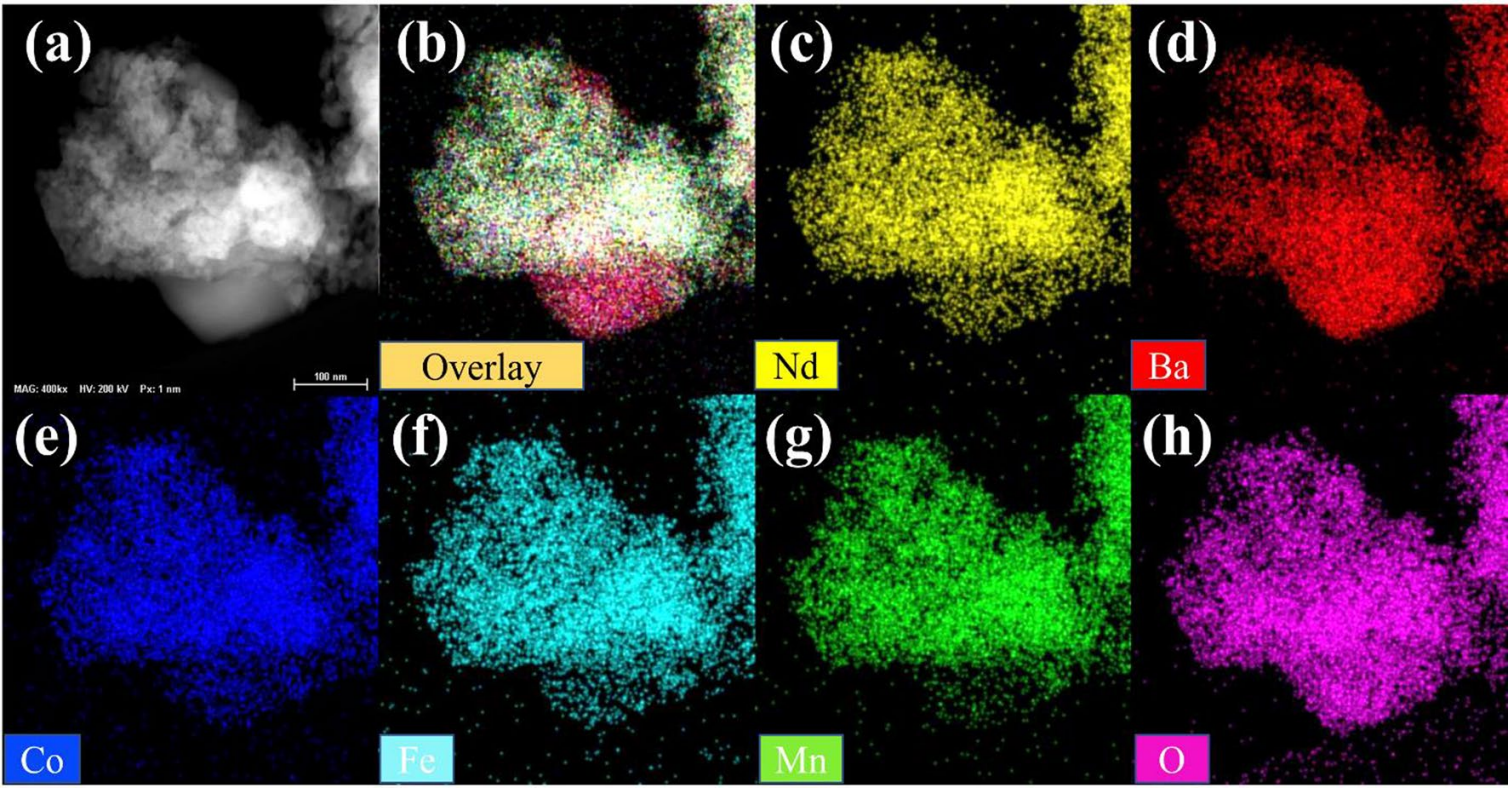
(**a**) TEM- HAADF image and STEM-EDS mapping of NBCFM sample (**b**) overlay (**c**) Nd, (**d**) Ba, (**e**) Co, (**f**) Fe, (**g**) Mn, and (**h**) O.

Figure 2a–c shows the TEM images of BSCF, PBCO, and NBCFM. Spherical and rectangular perovskite morphologies are detected after ball milling. Because the perovskite particles are relatively small after ball milling, they provide sufficient active sites owing to their high surface area and channels, which facilitate mass transport during electrochemical reactions^14^. Figure 3 shows the EDS mapping of NBCFM, which indicates a consistent and uniform distribution of Nd, Ba, Co, Fe, Mn, and O. In addition, a homogeneous distribution is revealed for BSCF and PBCO (Figs. S4, S5).

Techniques to improve the number of oxygen-deficient sites have been investigated as an effective strategy to improve the electrocatalytic performance of perovskites. However, the performance of this particular perovskite material is still mediocre compared with those of commercial Pt/C electrocatalysts. Therefore, composite or hybrid perovskites with carbon-based materials are required. Several types of carbon, such as graphene^21^, carbon nanotubes (CNTs)^22^, and acetylene black^23^ have been used as support materials to enhance the electrocatalytic performance of perovskites. The addition of carbon is acknowledged to improve the poor electrical conductivity of perovskite and provide impregnable adhesion to perovskite particles. In addition, the hybrid perovskite-carbon has a positive effect because carbon has a large surface area and favorable electrical conductivity. The structural modification of carbon by doping nitrogen atoms and reducing the oxygen content via the modified Hummer method is a known standard method to enhance the electrocatalytic performance of carbon^11,24,25^.

**Figure 4:**
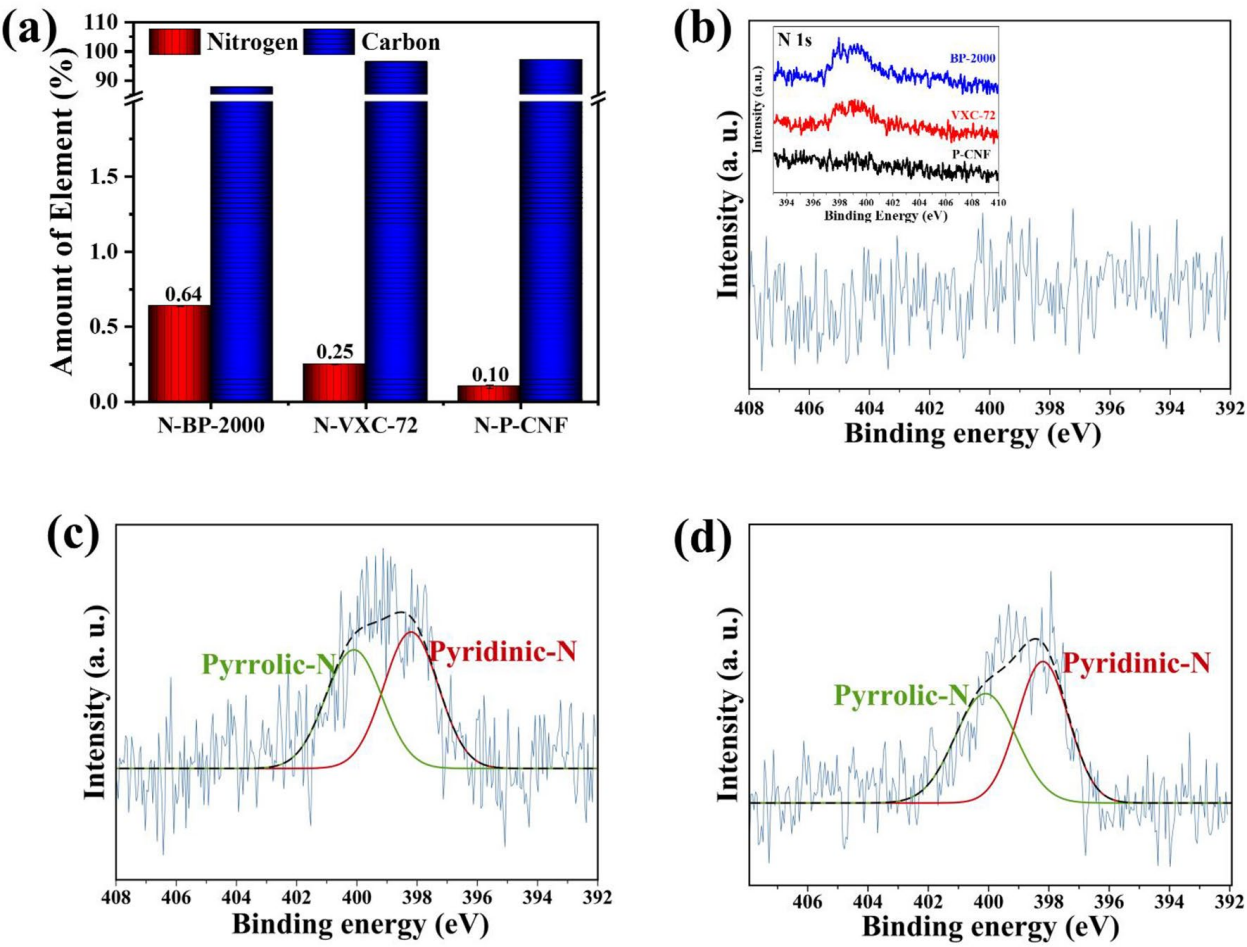
(**a**) EA and XPS N 1*s* spectra of (**b**) N-P-CNF (**c**) N-VXC-72 (**d**) N-BP-2000. Inset of (**b**) shows the XPS N 1*s* spectra comparison of various N-doped carbon.

Several types of carbon material underwent the nitriding treatment at 900 °C for 1 h under a N_2_–NH_3_ mixed gas (5.01 mol% NH_3_ and N_2_ balance) to enhance the electrocatalytic performance of carbon; the modified Hummer method treatment was not used because it produces toxic emissions^19^. The N-doped carbon was examined by EA to measure the nitrogen and carbon content. Figure 4a. shows that N-doped BP-2000 (N-BP-2000) has the highest nitrogen content of 0.64 wt%. The N-doped VXC-72 (N-VXC-72) and N-doped P-CNF (N-PCNF) have lower nitrogen contents of 0.25 and 0.10 wt%, respectively. Nitrogen is abundant in N-BP-2000, indicating that the nitrogen doping level is much higher than that of other carbons. According to Evlashin et al., the larger the carbon surface area, the greater the possibility of impingement of nitrogen and oxygen^26^. This was investigated as shown in Fig S3; among the carbon materials, BP-2000 (1455.68 m^2^ g^−1^) has a considerable BET surface area compared with those of VXC-72 (272.34 m^2^ g^−1^) and P-CNF (58.89 m^2^ g^−1^).

We also performed XPS measurements to study the electronic structure of nitrogen in N-doped carbon. As shown in the inset Fig. 4b, the comparison of the high-resolution spectrum of the N 1*s* peak at 400.0 eV shows that the N-P-CNF has no peak compared with N-VXC-72 and N-BP-2000. This is consistent with the EA analysis that P-CNF has negligible nitrogen content, and the N 1*s* spectrum does not recognize any peaks of nitrogen incorporated with carbon. In contrast, Fig. 4c,d show that the deconvolution of the N 1*s* spectrum of N-VXC-72 and N-BP-2000 consists of two peaks at 398.2 eV and 400.1, which are attributed to pyridinic-N and pyrrolic-N, respectively. Additionally, in our synthesized N-doped carbon samples, the nitrogen content was too low, and as a result, we did not observe the existence of peaks associated with quaternary N and some oxidized N, which is commonly observed in nitrogen-doped carbon. Pyridinic-N is known to possess N-type carbon structure that is most responsible for electrochemical properties. The result of the nitriding process of the carbon was preliminary evidence that there were sufficient active sites for the better performance of the ORR. Furthermore, the electrical conductivities of the perovskite and N-doped carbon were investigated. As shown in Fig. S6, the electrical conductivity of all the perovskite materials is almost zero at ambient temperature, with BSCF, PBCO, and NBCFM having values of 5.9 × 10^–30^, 8.5 × 10^–31^, and 7.8 × 10^–30^ S cm^−1^, respectively. The lack of electrical conductivity in perovskite materials is an intrinsic characteristic of metal oxides. In contrast, N-doped carbon materials have a relatively higher electrical conductivity than that of perovskite. The electrical conductivity measurement indicates that N-VXC-72 has the highest electrical conductivity (31.7 S cm^−1^), followed by BP-2000 (14.8 S cm^−1^), and P-CNF (11.5 S cm^−1^).

Pristine and N-doped carbon-perovskite hybridization was performed during the ink preparation for the RDE measurement. No heat treatment or physical mixing process was used in the hybridization process, which is common for perovskite-carbon hybridization experiments^11,14^. The carbon content was set to 20 wt% of the total catalyst ink dispersion. With this experimental design, the phase interaction between carbon and perovskite is easier to deduce. Figure 2d–f shows the TEM images of NBFCM with N-doped carbon. The interaction between the boundaries of carbon and perovskite tends to create inadequate bonding structures owing to less heat or physical treatment manipulation. Weak bonding was reported for better understanding during the investigation of the ORR mechanism of hybrid perovskite-carbon, and will be discussed later.

**Figure 5:**
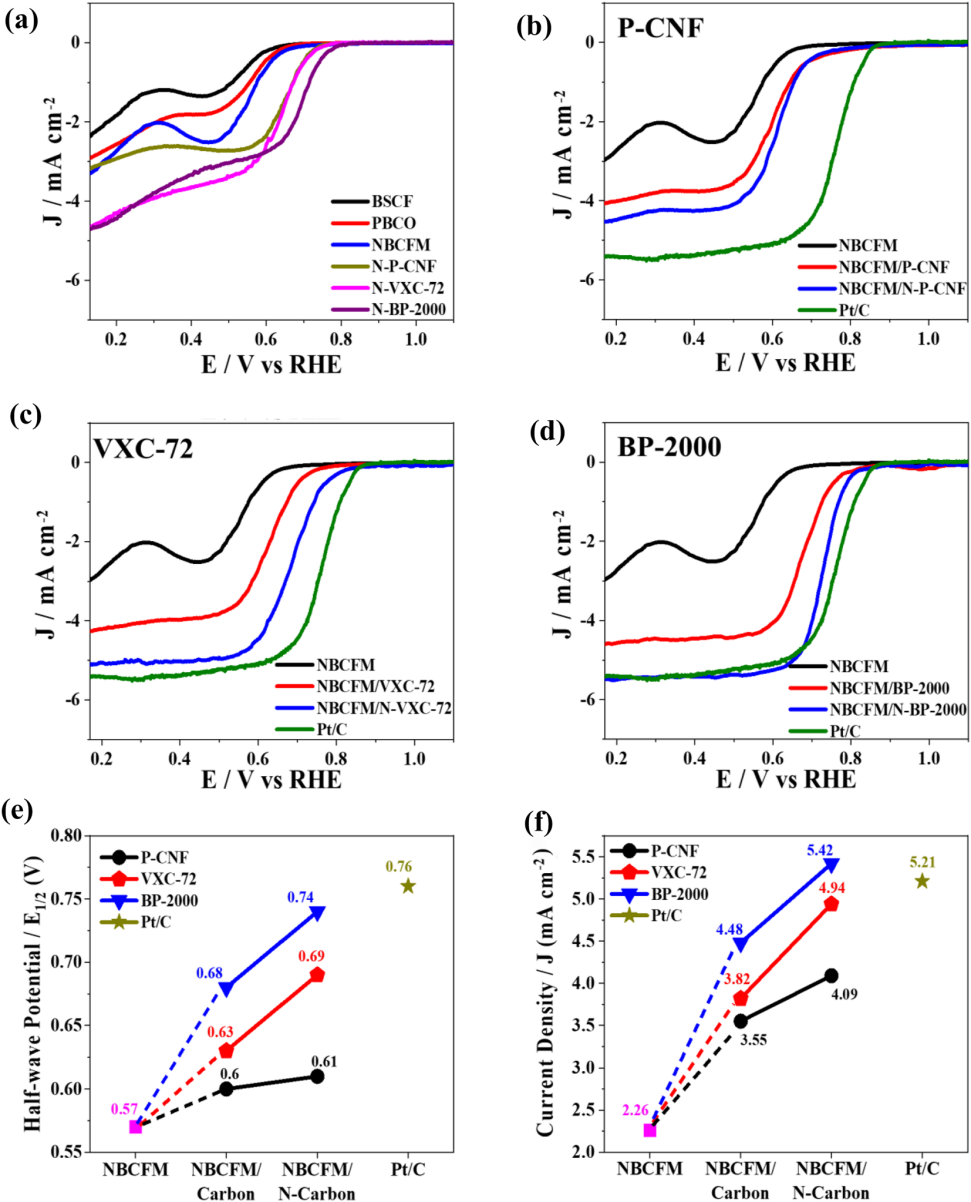
Comparison of electrochemical analysis of perovskites. (**a**) LSV curves of BSCF, PBCO, NBCFM, and N-doped carbon materials. LSV curves of hybrid NBCFM with several types of carbon (**b**) P-CNF (**c**) VXC-72, and (**d**) BP-2000. Interpretation of addition of N-doped carbon effect on (**e**) half-wave potential and (**f**) current density at 0.5 V.

To evaluate the performance of perovskites prior to hybridization with carbon, the ORR activity of pure perovskite was compared with that of the hybridized perovskites in terms of the specific current density (i_ORR_) and half-wave potential (E_1/2_). The magnitude of i_ORR_ can be used to determine how fast the electrochemical reaction can occur. The i_ORR_ at 0.5 V was recorded to quantify the ORR performance. In addition, E_1/2_ is defined as the midpoint between the limiting and zero current on the LSV curve. A higher E_1/2_ corresponds to a lower overpotential to reach the specific current density and is the counterpart of a higher ORR performance^27^. Figure 5a illustrates the LSV curves of the pure perovskite and N-doped carbon. NBCFM has the highest E_1/2_ and i_ORR_ of 0.57 V and 2.26 mA cm^−2^, respectively, followed by PBCO (0.56 V and 1.56 mA cm^−2^), and the lowest is that of BSCF (0.54 V and 1.06 mA cm^−2^). The i_ORR_ of perovskite materials is relatively negligible owing to the poor ability of electrons to flow through the electrodes. This state is a general characteristic of metal oxides, including perovskites, following the Mott–Hubbard insulator theory which explains that the electrons localized on the d-levels of metal oxides cause electrons to be in a stabilized state^28^. The large band gap, crystal defects, and small particle sizes of metal oxides may also contribute to the insulating character of perovskite materials^29,30^.

Interestingly, as shown in Fig. 5a., the ORR performance of all the N-doped carbons exceeds that of the pure perovskite materials. Table S1 and Fig. S7a show that BP-2000 exhibits a notable improvement in E_1/2_ and i_ORR_ (from 0.63 to 0.69 V and 2.63 to 3.14 mA cm^−2^, respectively, corresponding to a percentage increase of 9.5 and 19.4%) compared with the other materials after the nitriding process. In comparison, the E_1/2_ and i_ORR_ of P-CNF, the carbon with the lowest nitrogen content, increases only by 6.4% from 0.62 to 0.66 V and 11% from 2.18 to 2.42 mA cm^−2^, respectively.

The trend in the ORR performance of the sample was consistent with that of the XPS and EA data, which showed that the nitrogen content and N-type carbon structure have a crucial effect on the performance of carbon. Figure S7b displays the CV characteristics of all the N-doped carbons. The CV curve of N-BP-2000 shows the largest pseudo-capacitance area compared with those of N-VXC-72 and N-PCNF. It is known that the pseudo-capacitance area of carbon corresponds to the specific surface area, electrical conductivity, and amount of redox-active nitrogen species^22,31^. This is consistent with the BET observation that BP-2000 has the largest surface area among other carbons. From the perspective of electrical conductivity and the concentration of redox-active nitrogen species, N-BP-2000 has moderate electrical conductivity and consists of pyridinic-N and pyrrolic-N. In contrast, owing to the lowest electrical conductivity, smallest surface area, and lack of redox-active nitrogen species, N-PCNF demonstrates a narrow pseudo-capacitance area. Figure S7c–e. illustrates the calculation of the Tafel slope from the LSV polarization curves at the low overpotential of all the N-doped carbons. N-BP-2000 has the smallest Tafel slope (83.7 mV dec^−1^) compared with those of N-VXC-72 (86.7 mV dec^−1^) and N-P-CNF (89.7 mV dec^−1^). The Tafel slope value suggests that N-BP-2000 has faster ORR kinetics than those of the perovskites, and the first electron transfer may be the rate-determining step (RDS) for the ORR in alkaline media^32,33^.

Further N-doped carbons, particularly the pyridinic-N-type, have been shown to improve the ORR performance. Pyridinic-N is a nitrogen species that binds with two carbon atoms, and it has been proven as an active site for carbon nitriding owing to its adsorption of O_2_^26,34,35^. Because nitrogen has a comparable atomic size to that of carbon and is more electronegative than carbon, it can be used as a doping agent^36^. Nitrogen can be doped into both basal and edge planes, creating strong covalent bonds and high stability. Nitrogen also has an extra valence electron compared with carbon, which increases the electronic density state near the Fermi level and makes it more electronically conductive by facilitating electron transfer from the electronic bands of the C to O_2_ σ* antibonding orbitals^37,38^.

P-CNF, VXC-72R, and BP-2000 were used to better understand the importance of carbon materials in hybrid perovskite-carbon. Pristine and N-doped carbon were mixed with perovskite, and the ORR activity was observed. The ORR performance of the hybrid perovskite-carbon was markedly improved compared with that of pure perovskite and carbon materials. As shown by the LSV curves in Fig. 5b–d, the ORR performance of NBCFM is much improved and reaches the performance of the commercial Pt/C electrocatalyst mainly when the perovskites were mixed with carbon; the performance improves markedly after N-BP-2000 is used as the tandem carbon. As shown in Fig. 5e–f, pure NBCFM has E_1/2_ and i_ORR_ values of 0.57 V and 2.26 mA cm^−2^, respectively. The performance of NBCFM/BP-2000 increases to 0.68 V and 4.48 mA cm^−2^, respectively, when hybridized with pristine BP-2000. Furthermore, N-BP-2000 increases the E_1/2_ and i_ORR_ of NBCFM/N-BP-2000 to 0.74 V and 5.42 mA cm^−2^, which are analogous to the Pt/C catalytic activities of 0.76 V and 5.21 mA cm^−2^. In addition, the enhancement of hybrid NBCFM with VXC-72 has a similar tendency to that of BP-2000, where the E_1/2_ and i_ORR_ of NBCFM/VXC-72 and NBCFM/N-VXC-72 increases to 0.63 V and 3.82 mA cm^−2^, and 0.69 V and 4.94 mA cm^−2^, respectively. In contrast, hybridization with pristine and N-P-CNF does not increase E_1/2_ and i_ORR_ sufficiently, possibly due to the intrinsic properties and nitrogen content of N-P-CNF. Figures S8a and S9a show the LSV curves of the hybrid BSCF and PBCO with different carbon types before (dashed line) and after (solid line) nitriding. An identical trend was observed in the ORR performance of both BSCF and PBCO. As shown in Figs. S8c–d and S9c–d, the E_1/2_ and i_ORR_ values of pure BSCF and PBCO are 0.50 V, 1.06 mA cm^−2^, and 0.56 V, 1.56 mA cm^−2^, respectively. E_1/2_ and i_ORR_ improved notably after mixing the pure BSCF and PBCO with N-BP-2000 to 0.72 V, 4.91 mA cm^−2^, and 0.72 V, 5.03 mA cm^−2^, respectively. The utilization of N-VXC-72 and N-P-CNF for BSCF and PBCO resulted in slightly lower E_1/2_ and i_ORR_ than that of N-BP-2000. Therefore, it can be concluded that the ORR performance is highly dependent on the type of carbon material and its N-doped content, where a higher nitrogen content will improve the ORR performance more than a lower one.

**Figure 6:**
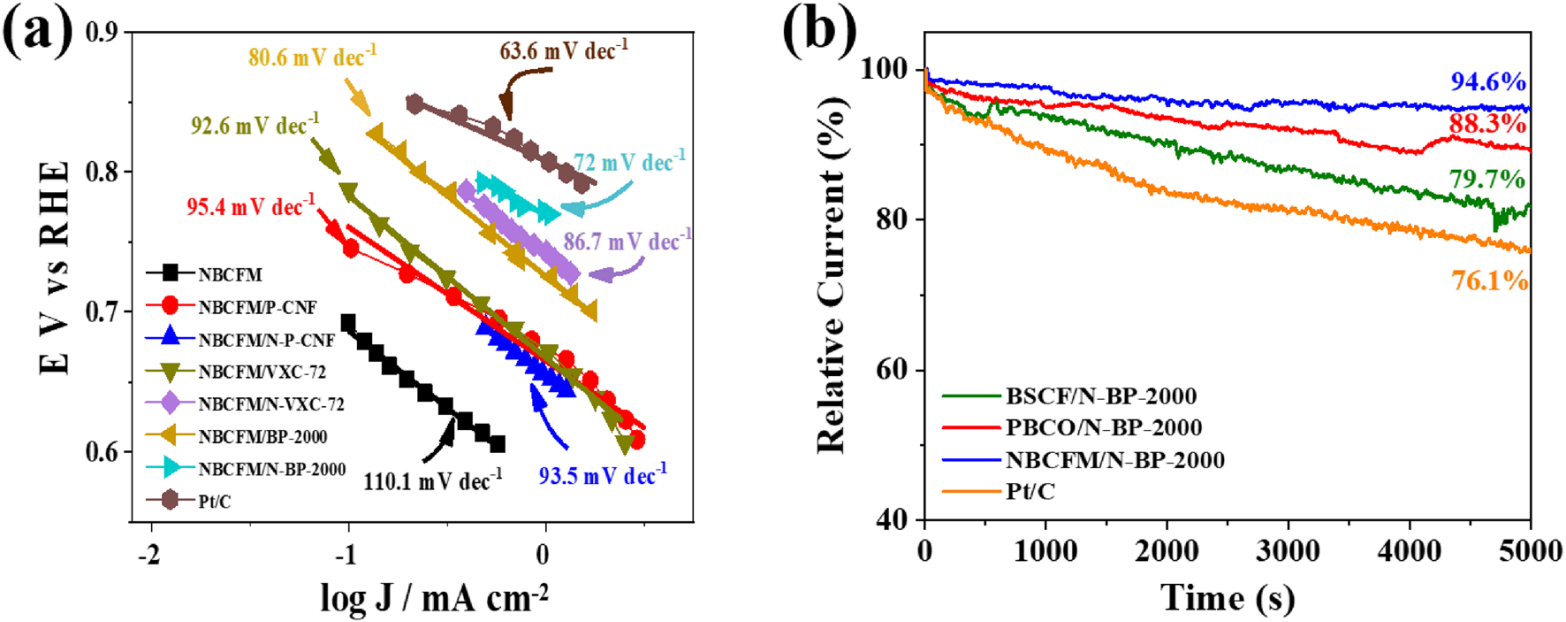
(**a**) Tafel slope of hybrid NBCFM with different carbon materials. (**b**) Stability test using chronoamperometry of the best performance of BSCF, PBCO and NBCFM hybridized with N-doped BP-2000 at 0.7 V, 1600 rpm in O_2_ saturated 0.1 M KOH.

To understand the ORR catalytic performance of all the hybrid perovskite-carbon catalysts, we examined the Tafel slope from the LSV curves. The Tafel slope of the hybrid NBCFM with various carbon materials is shown in Fig. 6a; the Tafel slopes of PBCO and BSCF are shown in Figs. S8b and S9b, respectively. Pure NBCFM, PBCO, and BSCF exhibit high Tafel slope values of 110.1, 112.3, and 169.7 mV dec^−1^, respectively, indicating that sluggish ORR kinetics occur on the pure perovskite material. In contrast, when perovskite is hybridized with carbon, the Tafel slope decreases according to the type of carbon used and its degree of nitridation. The lowest Tafel slope is achieved by hybridization with N-BP-2000. The Tafel slopes of BSCF/N-BP-2000 and PBCO/N-BP-2000 are 76.4 and 74.8 mV dec^−1^, respectively. NBCFM/N-BP-2000 has the lowest Tafel slope (72 mV dec^−1^) close to that of Pt/C (63.6 mV dec^−1^), indicating that the perovskite/N-BP-2000 has fast ORR kinetics comparable with that of Pt/C.

Furthermore, a stability test of the sample along with Pt/C was performed using chronoamperometry to observe the nature of the active site of the hybrid perovskite-carbon. Chronoamperometry was performed at 0.7 V with a rotation speed of 1600 rpm. As shown in Fig. 6b, after 5000 s, the hybrid NBCFM/N-BP-2000 is the most stable with a negligible decay of 5.4% in current density, while PBCO/N-BP-2000 and BSCF/N-BP-2000 are degraded considerably, by 11.7% and 20.3%, respectively. However, the stability of all hybrid perovskites comprising N-BP-2000 is superior to that of Pt/C, as the degradation is 23.9%. The excellent stability of the hybrid NBCFM/N-BP-2000 is attributed to the ability of NBCFM to inhibit the formation of the amorphous layer, especially the generation of Co species to become Co oxyhydroxide^11^. Furthermore, the alkaline media is known to generate Pt-OH species, and oxidation occurs on the surface of Pt/C, which causes the loss of the active site and excessively reduces the stability^39,40^.

Based on the above results and the ORR observation of pure perovskite, pristine and N-doped carbon, and the hybrid perovskite-carbon, we found that N-doped carbon plays a crucial role in the hybrid perovskite—carbon system. The N-doped carbon contributes to the enhancement of electrical conductivity and actively participates in the reduction of oxygen. Previous studies have postulated at least three theories of synergistic mechanisms for the hybrid perovskite-carbon interactions. The first theory is the ligand effect, where charge transfer may have occurred between carbon and perovskite^41^. The second mechanism is covalent bonding or a new phase being formed at the perovskite-carbon interface^42^. The spillover effect is the last mechanism proposed to explain the interaction between perovskite and carbon^14^. This mechanism emphasizes that carbon improves electrical conductivity and is more attractive because of the generation of OH^−^ from the reduction of O_2_^43^.

The design of the perovskite-carbon hybrid materials in this study involved little external treatment. The mixing process was performed while preparing the catalyst ink dispersion for the ORR evaluation without any mechanical or heat treatment process. This was also proved by observing the morphological surface interface between perovskite and carbon, as shown in the TEM images in Fig. 2d–f. Clearly, the surface interaction does not produce a specific new phase region. Thus, the possibility of the formation of a new phase at the interface is unlikely. In addition to the ligand effect, the measurement of the ORR performance of carbon (shown in Table S1) shows that the carbon materials not only contribute a conductivity effect in the hybrid perovskite-carbon but also exhibit a neglected ORR performance. Moreover, the hybridization of perovskite with a large surface area and a high degree of N-doped carbon is essential for accelerating the performance of perovskite materials as NPGM electrocatalysts.

## Conclusion

This study has explained the role of N-doped carbon in hybrid perovskite-carbon materials. The N-doped carbon was a critical element in the production of ORR performances comparable with those of commercial Pt/C electrocatalysts. The excellent ORR performances may also be attributed to the pyridinic-N site in the carbon material, which resulted from the nitriding process. The carbon atom next to the pyridinic-N acted as an O_2_ adsorbent and accelerated oxygen reduction. Furthermore, another vital contribution of the carbon material in the hybrid perovskite material was the improvement of electron conductivity. Therefore, we may conclude that for the development of NPGMs based on perovskite material, the carbon treatments are equally important to the treatment of perovskite material itself. Based on our findings, we conclude that the selection of carbon material and its treatment would determine the sustainability of hybrid perovskite-carbon as an affordable, stable, and applicable ORR electrocatalyst.

### Supplementary Information

Supplementary Information.

## Data Availability

The datasets used and/or analysed during the current study available from the corresponding author on reasonable request.

## Abbreviations

CA: Citric acid
CV: Cyclic voltammograms
EA: Elemental analysis
EG: Ethylene glycol
GC: Glassy carbon
LSV: Linear sweep voltammograms
NPGM: Non-platinum group metal
PEFC: Polymer electrolyte fuel cells
RDE: Rotating disk electrode
RDS: Rate-determining step
