# Supplementary material for: Synergistic effect of perovskites and nitrogen-doped carbon hybrid materials for improving oxygen reduction reaction
Source: Sci Rep. 2023 Nov 13;13:19832. doi: 10.1038/s41598-023-47304-4 (PMC10645751; doi:10.1038/s41598-023-47304-4)
Supplement: Supplementary file 1 — Supplementary Information. [file 41598_2023_47304_MOESM1_ESM.docx]

# Supporting information

**Synergistic Effect of Perovskites and Nitrogen-Doped Carbon Hybrid Materials for Improving Oxygen Reduction Reaction**

R. Rohib,^a,b^ Saeed Ur Rehman,^a^ Eunjik Lee,^a*^ Changki Kim,^a^ Hyunjoon Lee,^a^ Seung-Bok Lee,^a^ Gu-Gon Park^a,b^^*^

^a^Fuel Cell Laboratory, Korea Institute of Energy Research (KIER), 152 Gajeong-ro, Yuseoung-gu, Daejeon, 34129, Republic of Korea

^b^Department of Energy Engineering, University of Science and Technology, 217 Gajeong-ro, Yuseong-gu, Daejeon 34113, Republic of Korea

*Corresponding author: [ejlee21@kier.re.kr](mailto:ejlee21@kier.re.kr) (Eunjik Lee), [gugon@kier.re.kr](mailto:gugon@kier.re.kr) (Gu-Gon Park)

**
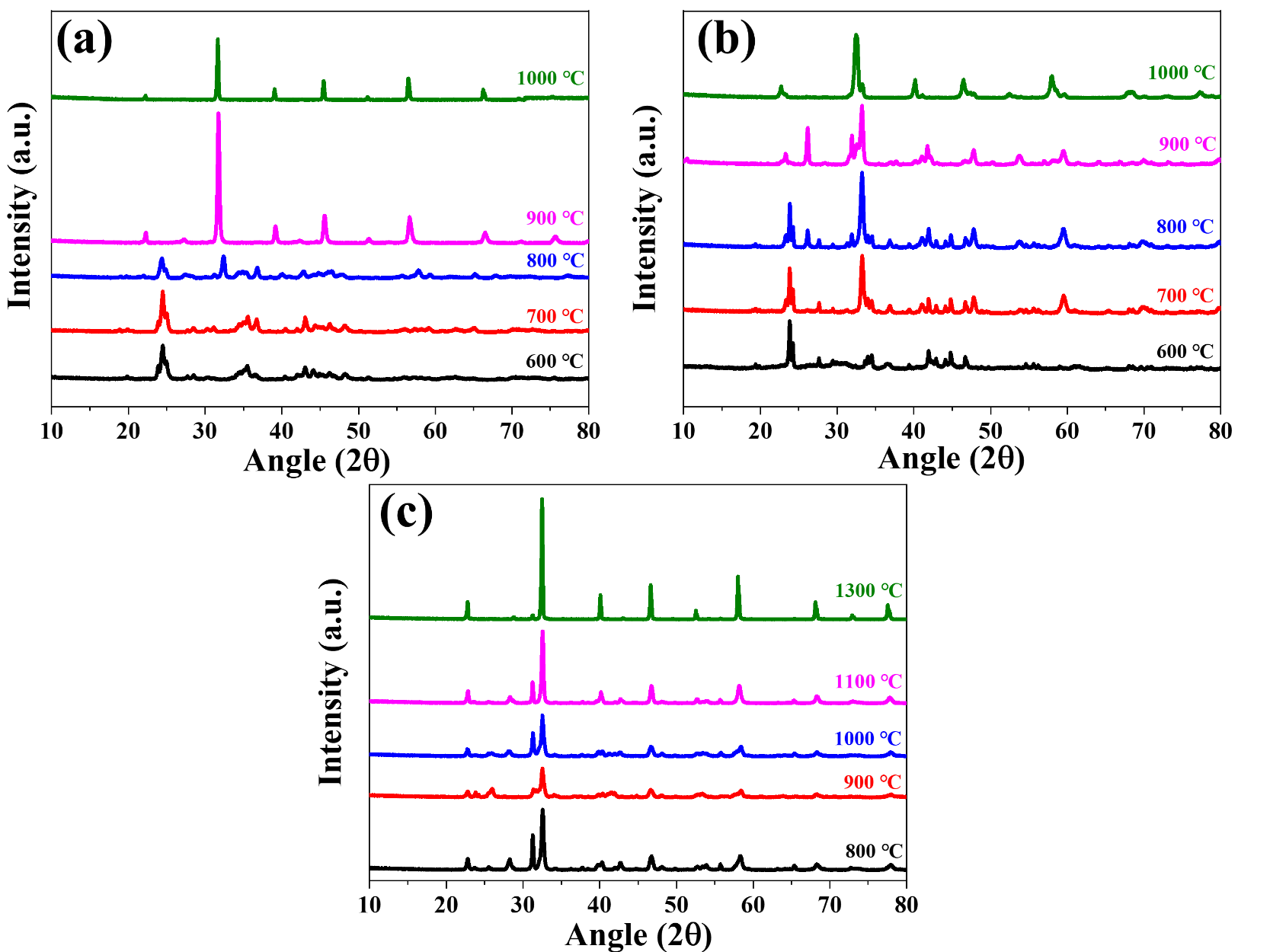
Figure S1.** XRD patterns of (a) BSCF, (b) PBCO, (c) NBCFM at different heat treatment temperatures

**
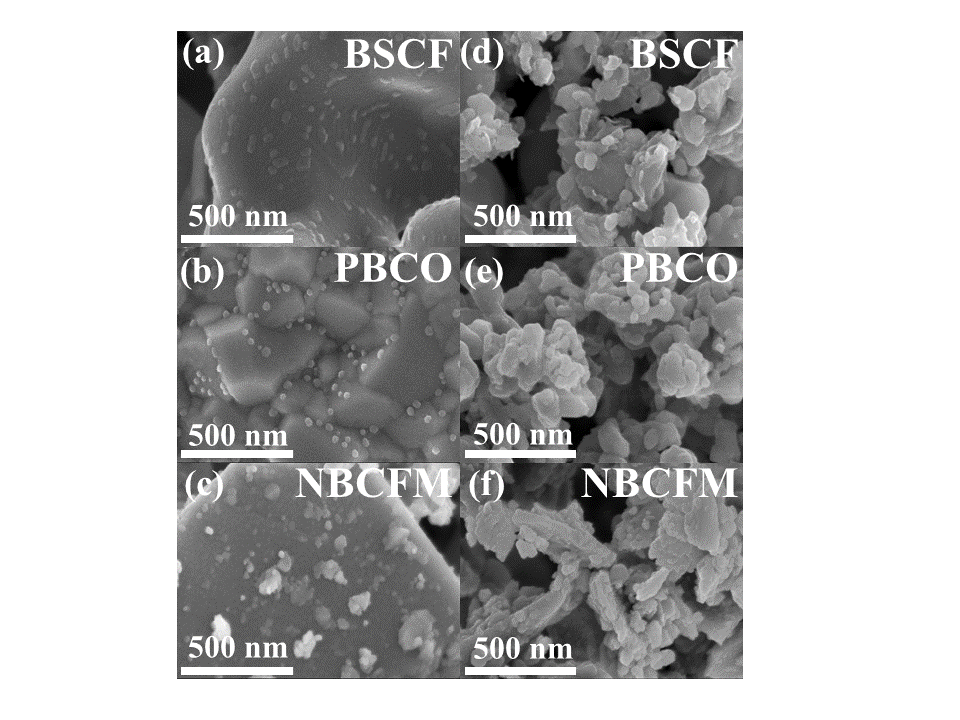
Figure S2**. SEM images of perovskite materials (a-c) before and (d-f) after ball mailing. The agglomerated structure with micrometer size successfully reduced became 10 - 100 nm by ball milling process


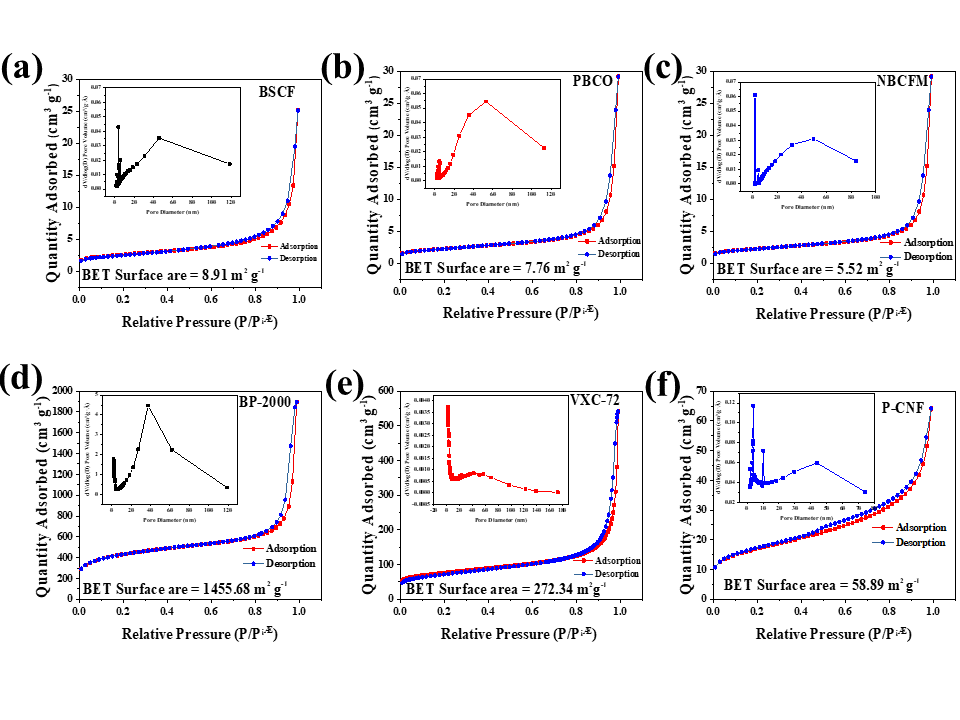
**Figure S3.** BET analysis of nitrogen adsorption-desorption isotherm curve and its pore size distribution (a) ball-milled BSCF, (b) ball-milled PBCO, (c) ball-milled NBCFM, (d) BP-2000, (e) VXC-72 and (f) P-CNF samples.

**
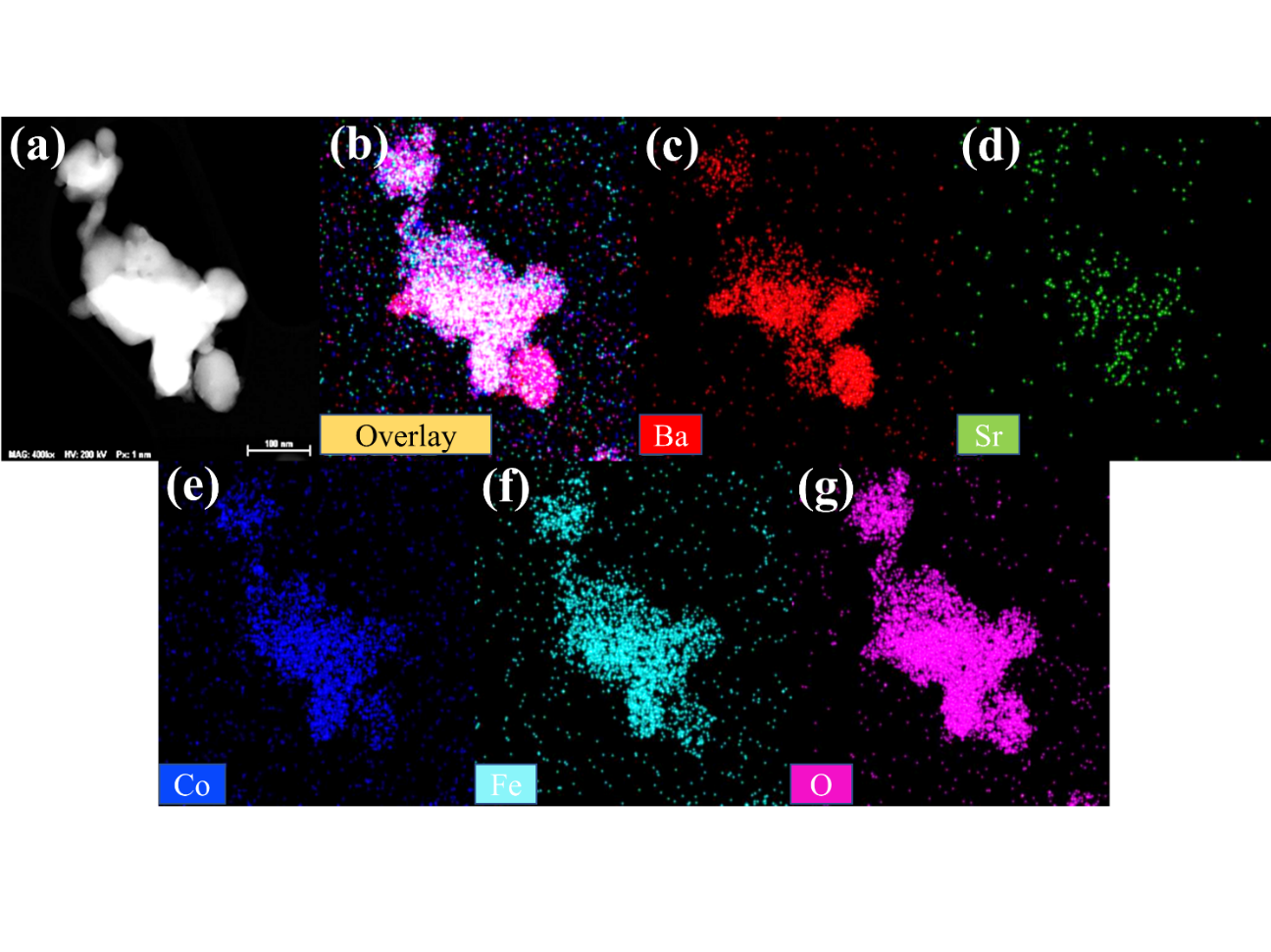
Figure S4*.*** (a) TEM- HAADF image and (b-g) TEM-EDS mapping of BSCF sample

*
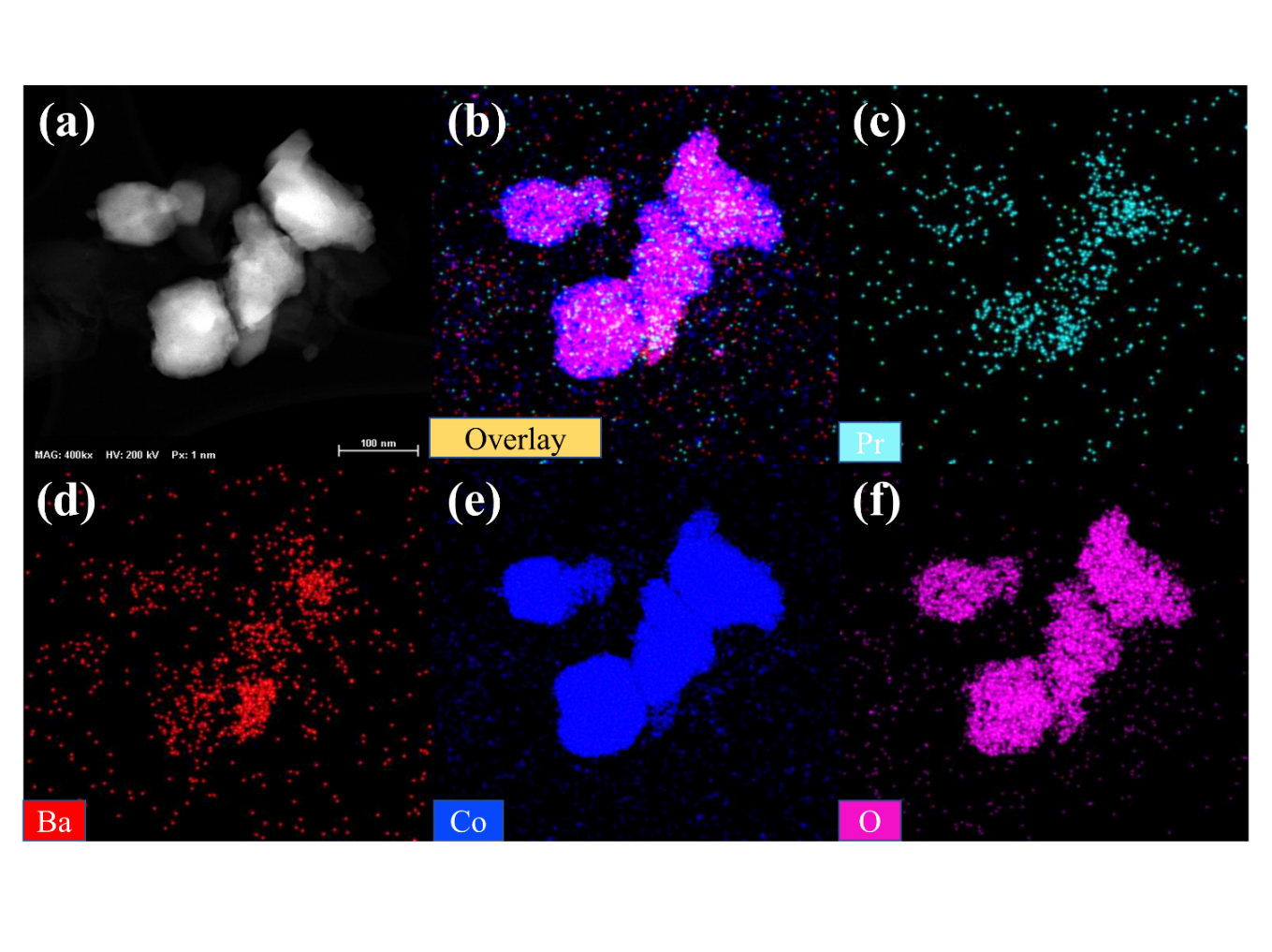
*

**Figure S5*.*** (a) TEM- HAADF image and (b-f) TEM-EDS mapping of PBCO sample

**
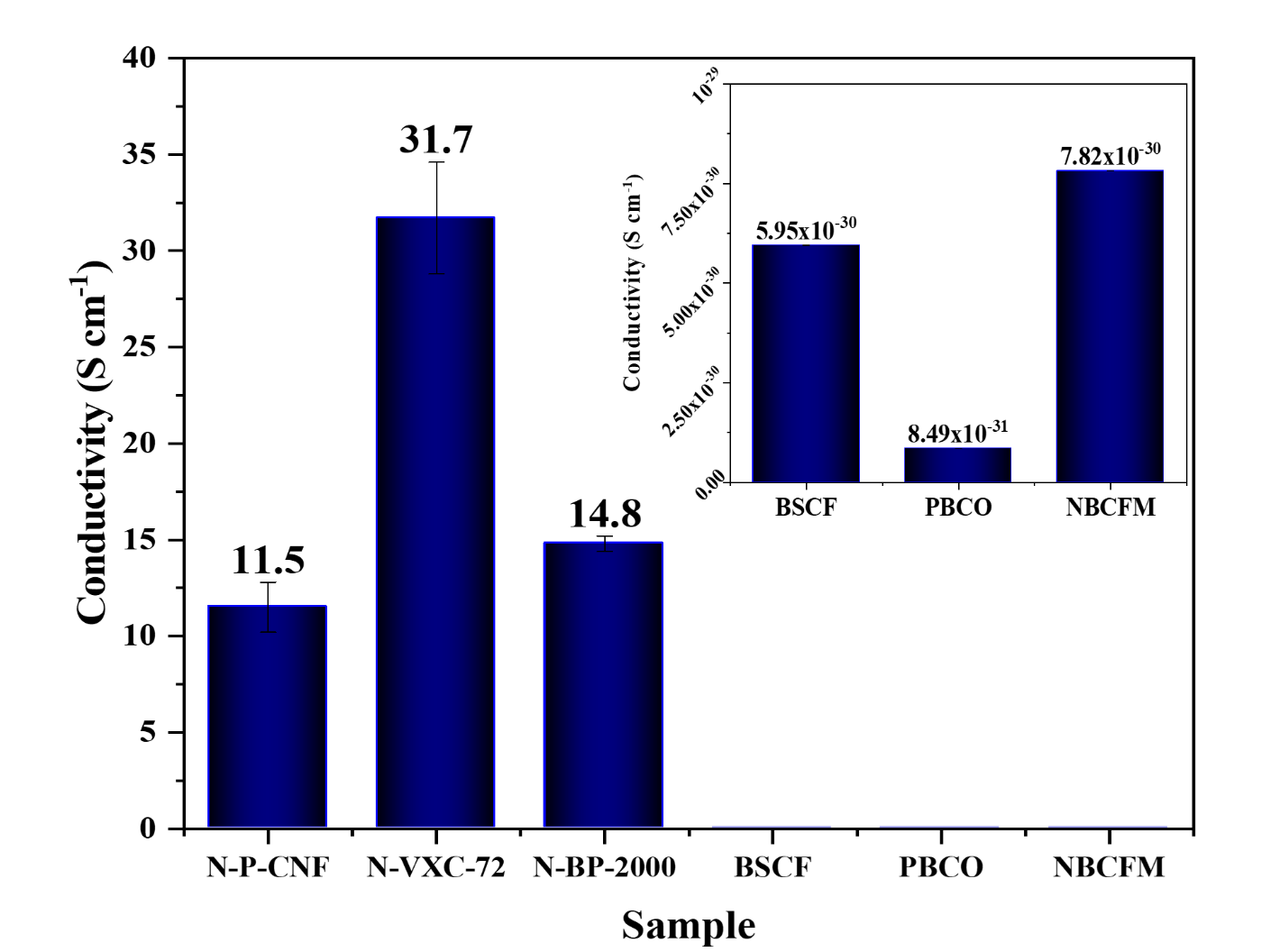
Figure S6.** Comparison of powder electrical conductivity of perovskite and N-doped carbon materials. Inset images, the electrical conductivity of perovskite material in different scale bar

**
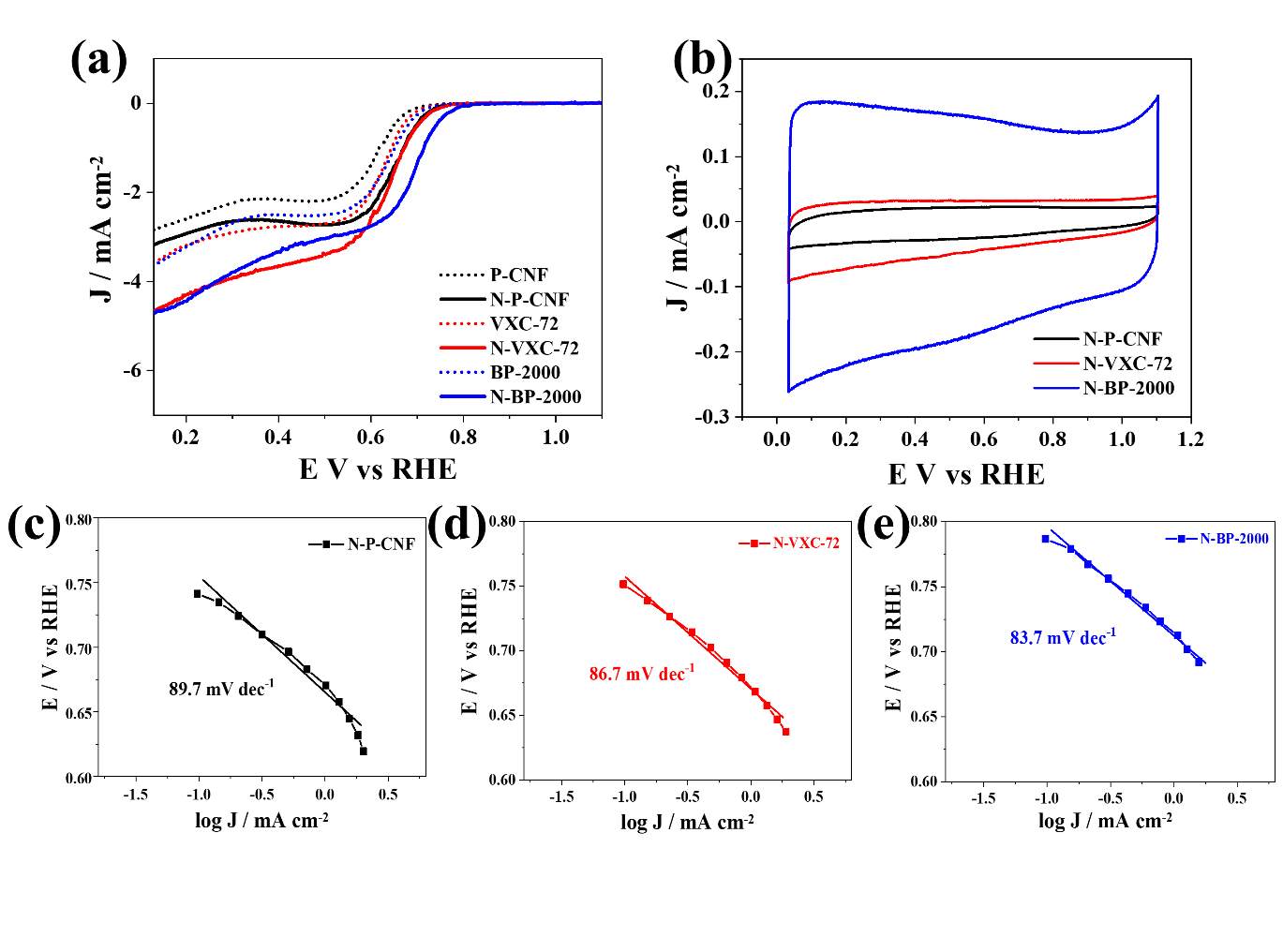
Figure S7.** a) The comparison of LSV curves of carbon before and after treatment at 900 °C for 1 h under NH_3_ environment. (b) The CVs curve of N-doped carbon shows the carbon capacitance correspond to the carbon surface area. Tafel slop plot obtained from LSV curves was calculated for (c) N-PCNF (d) N-VXC-72, and (e) N-BP-2000

**Table S1.** Electrochemical properties of carbon before and after heat treatment under NH_3_ and pure perovskite materials

| **Material** | **Half-wave potential**  **(V)** | | | | **Current Density**  **(mA cm^−2^)** | | | **Tafel Slope**  **(mV dec^−1^)** | |
| --- | --- | --- | --- | --- | --- | --- | --- | --- | --- |
|  | **Pristine** | **N-doped** | | **Improvement** | **Pristine** | **N-doped** | **Improvement** | **Pristine** | **N-doped** |
| P-CNF | 0.59 | 0.64 | 3.2% | | 2.18 | 2.42 | 11.0% | 97.5 | 89.7 |
| VXC-72 | 0.62 | 0.66 | 4.7% | | 2.71 | 3.39 | 25.1% | 84.3 | 86.7 |
| BP-2000 | 0.63 | 0.69 | 9.5% | | 2.63 | 3.14 | 19.4% | 86.8 | 83.7 |
| BSCF | 0.50 | - | - | | 1.06 | - | - | 169.7 | - |
| PBCO | 0.56 | - | - | | 1.56 | - | - | 112.3 | - |
| NBCFM | 0.57 | - | - | | 2.26 | - | - | 110.1 | - |

**
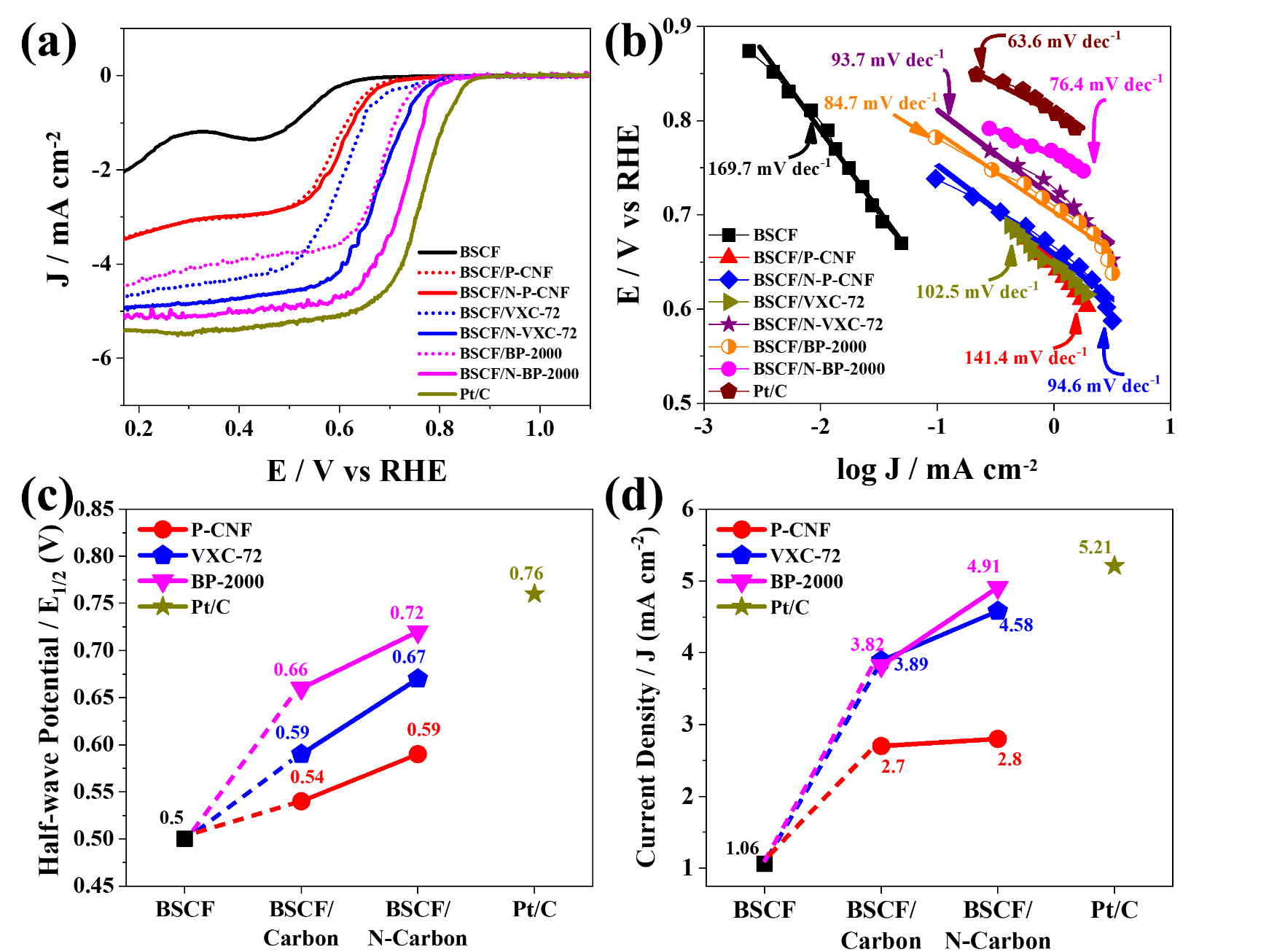
Figure S8.** Comparison of ORR polarization curves of pure BSCF and its hybrid using various type of carbon before and after nitriding process; dashed line (pristine carbon) and solid line (N-doped carbon) at the rotating speed 1600 rpm in 0.1 M KOH corresponding to b) Tafel slope. The interpretation of N-doped carbon addition effect on the c) half-wave potential and d) current density of hybridized BSCF.


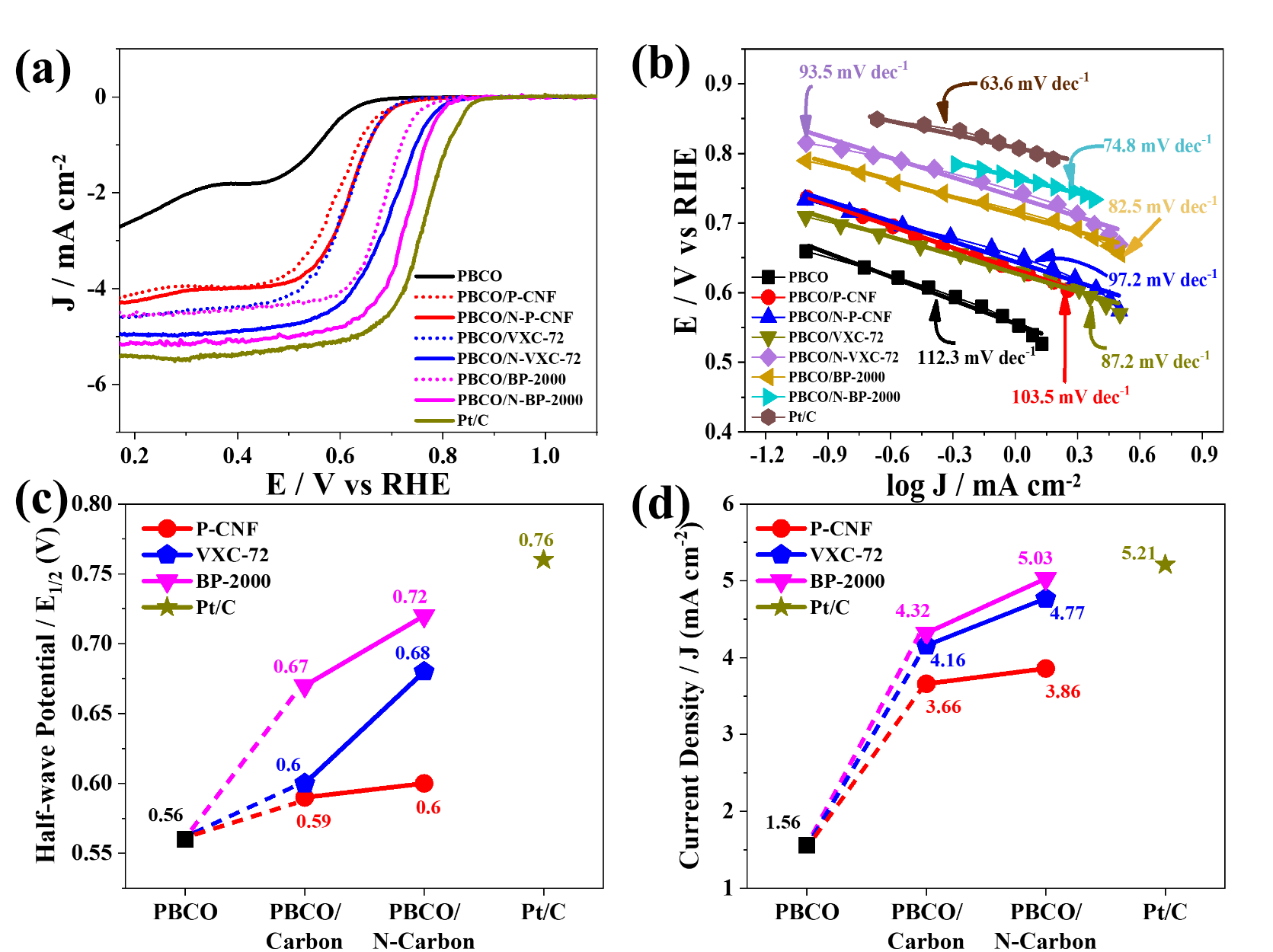
**Figure S9.** Comparison of ORR polarization curves of pure PBCO and its hybrid using various type of carbon before and after nitriding process; dashed line (pristine carbon) and solid line (N-doped carbon) at the rotating speed 1600 rpm in 0.1 M KOH corresponding to b) Tafel slope. The interpretation of N-doped carbon addition effect on the c) half-wave potential and d) current density of hybridized PBCO.
